# Clinical presentation of sporadic and hereditary pheochromocytoma/paraganglioma

**DOI:** 10.1530/EO-22-0040

**Published:** 2023-01-11

**Authors:** Sofia Maria Lider Burciulescu, Caren Randon, Frederic Duprez, Wouter Huvenne, David Creytens, Kathleen B M Claes, Robin de Putter, Guy T’Sjoen, Corin Badiu, Bruno Lapauw

**Affiliations:** 1 CI Parhon National Institute of Endocrinology, Bucharest, Romania; 2 Department of Thoracic and Vascular Surgery, Ghent University Hospital & Department of Human Structure and Repair, Faculty of Medicine and Health Sciences, UGent, Ghent, Belgium; 3 Department of Radiotherapy-Oncology, Ghent University Hospital, Ghent Belgium & Department of Human Structure and Repair, Faculty of Medicine and Health Sciences, UGent, Ghent, Belgium; 4 Department of Head and Neck Surgery, Ghent University Hospital & Department of Head & Skin, Faculty of Medicine and Health Sciences, UGent, Ghent, Belgium; 5 Department of Pathology, Ghent University Hospital, Ghent University & Department of Diagnostic Sciences, Faculty of Medicine and Health Sciences, UGent, Ghent, Belgium; 6 Center for Medical Genetics, Ghent University Hospital & Department of Biomolecular Medicine, Faculty of Medicine and Health Sciences, UGent, Ghent, Belgium; 7 Department of Endocrinology, Ghent University Hospital & Department of Internal Medicine & Pediatrics, Faculty of Medicine and Health Sciences, UGent , Ghent, Belgium; 8 Carol Davila University of Medicine and Pharmacy, Bucharest, Romania

**Keywords:** pheochromocytoma, paraganglioma, multiple endocrine neoplasias, neuro-endocrine tumours

## Abstract

Pheochromocytomas (PHEO) and paragangliomas (PGL) can occur sporadic or within genetic predisposition syndromes. Despite shared embryology, there are important differences between PHEO and PGL. The aim of this study was to describe the clinical presentation and disease characteristics of PHEO/PGL. A retrospective analysis of consecutively registered patients diagnosed with or treated for PHEO/PGL in a tertiary care centre was performed. Patients were compared according to anatomic location (PHEO vs PGL) and genetic status (sporadic vs hereditary). In total, we identified 38 women and 29 men, aged 50 ± 19 years. Of these, 42 (63%) had PHEO, and 25 (37%) had PGL. Patients with PHEO presented more frequently with sporadic than hereditary disease (45 years vs 27 (77%) vs 8 (23%)) than patients with PGL (9 (36%) vs 16 (64%), respectively) and were older at diagnosis (55 ± 17 vs 40 ± 18 years, *P* = 0.001), respectively). About half of the cases in both PHEO and PGL were diagnosed due to disease-related symptoms. In patients with PHEO, tumour diameter was larger (*P* = 0.001), metanephrine levels higher (*P* = 0.02), and there was more frequently a history of cardiovascular events than in patients with PGL. In conclusion, we found that patients with PGL more frequently have a hereditary predisposition than those with PHEO, contributing to the fact that diagnosis is generally made earlier in PGL. Although diagnosis in both PHEO and PGL was mostly due to related symptoms, patients with PHEO more often presented with cardiovascular comorbidities than those with PGL which might relate to a higher number of functionally active tumours in the former.

## Introduction

Pheochromocytomas and paragangliomas (PPGL) are rare neuroendocrine tumours of the autonomic nervous system, embryologically originating from the neural crest. While pheochromocytomas (PHEO) arise in the adrenal medulla, paragangliomas (PGL) can occur at various body sites and are traditionally divided into head-and-neck PGL (HNPGL) and PGL located in the thorax, abdomen, or pelvis. The incidence of PPGL is about 0.7 cases per 100,000 persons/year (Leung *et al.* 2021). The functionality of PHEO is defined by epinephrine, norepinephrine, and/or dopamine overproduction, whereas PGL can only excrete norepinephrine and/or dopamine (except Zuckerkandl organ PGL which secretes epinephrine). Also, some PPGLs are non-functional (most of HNPGLs). These differences in catecholamine secretion patterns can generate a different intensity of symptoms, but in general, most patients with PPGL present with non-specific complaints. Together with increasing use of medical imaging, this leads to an important percentage of incidentally discovered PPGL (~50%) (Gruber *et al.* 2019).

Importantly, up to 40% of patients with a PPGL have a hereditary predisposition (Lenders *et al.* 2020) – the highest heritability rate among human tumours. Major suggestive features of hereditary PPGL are familial history of the disease, bilateral cancers affecting paired organs, multiple primary PPGL in the same individual, recurrent or malignant disease, and early age of onset (i.e. <45 years old) (Amar *et al.* 2005, Lenders *et al.* 2014). This high percentage of hereditary PPGL makes genetic testing to identify patients and relatives at risk to (re)develop disease and thus enable tumour screening a key step in the management of these rare diseases. Although algorithms for targeted genetic testing might become redundant due to the increased efficiency and lower cost of next-generation sequencing (Ben Aim *et al.* 2019, Lenders *et al.* 2020), research on differences in disease presentation between sporadic and hereditary PPGL as well as on possible genotype–phenotype correlations in hereditary PPGL still is important for better surveillance and outcome prediction in patients and thus an important basis for future personalized therapy (Crona *et al.* 2019, Main *et al.* 2020).

In this study, we investigated the clinical presentation and disease characteristics of sporadic and hereditary PPGL and evaluated the applicability of genotype–phenotype correlations described in the algorithms for genetic screening in a cohort of Belgian patients.

## Materials and methods

A retrospective analysis of electronic medical records of 67 consecutively registered patients diagnosed and/or treated with PPGL in the Ghent University Hospital (Belgium) between 2002 and 2020 was performed. Patients were identified through patient registries of the departments of Pathology, Surgery, and Endocrinology. Patients were divided according to anatomic location (PHEO vs PGL) and according to genetic status (hereditary vs sporadic PPGL; patients without genetic testing results (*n* = 7, all with PHEO) were not included in these latter analyses). General characteristics (age at diagnosis, gender, anthropometric parameters), method of discovery, clinical phenotype (symptoms, functionality, localization, presence of multiple primary tumours at diagnosis, malignancy (metastases or tumour recurrence), outcome) and hormonal phenotype (metanephrine, normetanephrine and dopamine levels), secretion pattern, and tumour diameter (assessed with CT/MRI scan) were compared between patients with sporadic vs hereditary PHEO/PGL.

Pathway to discovery was categorized as incidental/through familial genetic screening or symptomatic (i.e. due to symptoms related with catecholamine overproduction or local complaints) discovery. Symptoms were grouped into classic triad (headache, palpitations, sweating), non-specific symptoms (hypertension, paroxysms, weight loss, anxiety, vomiting, syncope), tumour location-related complaints (dysphagia, tinnitus, deafness, vertigo, bone pain), and cardiovascular (CV) events (myocardial infarction (MI), Takotsubo cardiomyopathy, arrhythmias, or stroke.) Patients were defined to have multiple tumours at diagnosis if they presented with bilateral disease or distant synchronous tumours. PPGL were considered functional if either plasma or urinary fractionated (nor)metanephrine levels were >1.5 × upper limit of the respective normal (ULN) reference ranges. A noradrenergic secretion pattern was defined as predominant increases of normetanephrine accompanied by metanephrine concentrations <1.5 ULN, whereas an adrenergic secretion pattern was defined as increments >1.5 ULN for both metabolites.

Patients were genetically tested for germline pathogenic variants by ‘sequence by synthesis’ or direct sequencing technology, performed at the Center for Medical Genetics, Ghent University Hospital, Belgium, using KAPA HyperCap technology (Roche Diagnostics) or other depending on the diagnosis date. The gene panel included the most frequently mutated genes in PPGL: *RET, VHL, NF1, MAX, SDHA, SDHB, SDHC, SDHD, SDHAF2,* and *TMEM127*. Depending on phenotypic features and/or year of diagnosis, patients were tested for a variable number of these genes. For patients with hereditary and apparently hereditary phenotype (metastatic disease/multiple tumours at diagnosis), we evaluated the applicability of genotype–phenotype correlations, suggested in the algorithm for genetic screening (Lenders *et al.* 2014), to which we added SDHB immunohistochemistry results. This study was approved by the Ethical Review Board of Ghent University Hospital (BC-10125) and conducted in accordance with the Declaration of Helsinki.

All statistical procedures were performed using Statistical Package for Social Sciences 23.0 software package (SPSS Inc.). Continuous variables were described in terms of mean ± s.d. if their distribution was normal according to the Kolmogorov–Smirnov test and in terms of median and range, otherwise. For statistical analysis, we used non-parametric correlations, Kruskal–Wallis test, Fisher test, and Student’s *t*-test where appropriate. A two-sided *P*-value < 0.05 was considered to indicate statistical significance.

## Results

### Patient and PPGL characteristics

We identified 67 patients (38 women and 29 men) who were diagnosed and/or treated with a PPGL in our centre between 2002 and 2020. At diagnosis, 42 (63%) patients had PHEO (2 with bilateral tumours) and 25 (37%) had PGL (6 with multiple tumours), and the mean age was 50 ± 19 years (range 13–85 years). Sixty patients were genetically tested, of whom 24 (40%) presented with hereditary disease and 36 (60%) in whom no pathogenic mutation could be identified. In order of frequency, the mutant genes discovered in our cohort were *SDHD* (*n* = 10; 41.7% of all hereditary cases), *SDHB* (8; 33.3%), *VHL* (2; 8.3%), *RET* (2; 8.3%), *MAX* (1; 4.2%), and *NF1* (1; 4.2%). Patients with PHEO presented more frequently with sporadic than hereditary disease (27 vs 8), while in patients with PGL, the opposite was found (9 vs 16, respectively). The general and tumour characteristics of both groups are given in Table 1. The mean age at diagnosis was higher in patients presenting with PHEO vs those with PGL (55 vs 40 years, respectively, *P* < 0.001). Pathways to discovery were similarly distributed in both groups with half of the patients diagnosed due to symptoms (PHEO: 21 (50%); PGL: 13 (52%)) and the other half incidentally (*n* = 27)/due to genetic screening (*n* = 6) (PHEO: 21 (50%); PGL: 12 (48%)). Patients with PGL did present more often with tumour location-related symptoms (PHEO: 1 (2.3%); PGL: 7 (28%); *P* = 0.009). In our cohort, patients were already known with the following CV events at diagnosis of PPGL: MI (PHEO: 8; PGL: 1), Takotsubo cardiomyopathy (PHEO: 2; PGL: 2), arrhythmias (PHEO: 3; PGL: 0), and stroke (PHEO: 1; PGL: 0). Overall, prevalent CV events were more frequent in patients presenting with PHEO than in those with PGL (PHEO: 14 (33.3%); PGL: 3 (12%); *P* = 0.022); all patients with a history of CV events had functionally active disease.

**Table 1:** Patient and tumour characteristics in PHEO vs PGL. Data are presented as mean ± s.d., number (percentages), or median (absolute range).

*n* = 67	PHEO (*n* = 42)	PGL (*n* = 25)	*P* value
Age (years)	55 ± 17	40 ± 18	<0.001
Sex	25 F, 17 M	13 F, 12 M	0.615
BMI (kg/m^2^)	26.2 ± 4.7	25.1 ± 5	0.453
Localization			NA
	22 (52.5%) Right	12 (48%) HNPGL	
	18 (42.8%) Left	1 (4%) Thoracic	
	2 (4.7%) Bilateral	4 (16%) Abdominal	
		4 (16%) Pelvic	
		2 (8%) HNPGL + Thoracic	
		1 (4%) HNPGL+ Abdominal	
		1 (4%) Abdominal + Pelvic	
Functionality^a^	40/40 (100%)	10/17 (59%)	<0.001
Multiple tumours at diagnosis	3 (7.1%)	6 (24%)	0.069
Metastatic disease	4 (9.5%)	2 (8%)	0.650
Pathway to discovery			
Symptomatic	21 (50%)	13 (52%)	0.927
Incidentally/genetic screening	19 (45.2%)/2 (4.7%)	8 (32%)/4 (16%)	0.480/0.152
Symptoms^a^			
Classic triad	15/37 (40.5%)	5/19 (26.3%)	0.579
Non-specific	15/37 (40.5%)	7/25 (28%)	0.812
Tumour location-related	1/42 (2.3%)	7/25 (28%)	0.009
CV events	14 (33.3%)	3 (12%)	0.022
MN levels (*n* < 350 µg/24 h)	631 (range 63–32,525)	162 (range 4–400)	<0.001
Number of patients with MN levels above ULN	20/36	1/15	<0.001
xULN^b^	1.8	0.46	<0.001
NMN levels (*n* < 650 µg/24 h)	1854 (range 326–18,813)	684 (range 126–8694)	0.034
Number of patients with NMN levels above ULN	25/36	4/15	0.010
xULN^b^	7.65	5.82	0.093
D levels (*n* < 500 µg/24 h)	272 (range 62–6263)	304 (range 203–741)	0.269
Number of patients with D levels above ULN	3/21	2/10	0.353
xULN^b^	5.8	1.3	0.840
Tumour diameter (mm)	44 (range 17–200)	30 (range 11–110)	0.016
Follow-up duration (years)	5 (range 0.6–26)	4 (range 1–34)	0.851
Outcome			NA
	37 (88%) NED	16 (64%) NED	
	2 (4.7%) Active disease 1 (2.3%) Recurrence	7 (28%) Active disease 2 (8%) Recurrence	
	3 (7.1%) New disease	1 (4%) New disease	
	2 (4.7%) Deceased	3 (12%) New disease + recurrence	
		2 (8%) Deceased	
SDHB staining (− negative; + positive; +/− weak)	12 +	3 +	
	9 −	8 −	
	2 +/−	2 +/−	
	19 Not known	12 Not known	

^a^Data only available for subset of patients; ^b^Calculated only for those with functional tumours.

D, dopamine; MN, metanephrine; NMN, normetanephrine; NA, not applicable; NED, no evidence of disease; xULN, times upper limit of normal.

All PHEO and about two-thirds of PGL were considered hormonally functional (PHEO vs PGL, *P* < 0.001). Most PHEO (20/39) had an adrenergic secretion pattern, a finding that was not so for PGL (1/10). Tumour diameter weakly correlated with metanephrine level in the PHEO group (*r* = 0.17, *P* < 0.005) but not in the PGL group. We also found higher normetanephrine levels (5885 vs 3089 µg/24 h, *P* = 0.001) and larger tumour diameter (51.4 vs 28.6 mm, *P* = 0.02) in patients with a history of CV event vs those without. We did not find any relation between pathway to diagnosis and (nor)metanephrine levels or tumour diameter.

All patients with PHEO underwent surgery, except one patient who has large vessel transposition, which justifies improbability of the surgery. Four patients with PHEO-metastatic disease, after surgery, underwent MIBG (metaiodobenzylguanidine) therapy (3) and radiotherapy (1).

Most patients with PGL underwent surgery, either because of presumed functionality, symptoms, or tumour growth, except five patients with HNPGL that were surgically inaccessible and were treated with radiotherapy.

### Sporadic vs hereditary PHEO

We did not identify important differences in general patient or tumour characteristics in patients with sporadic vs hereditary PHEO. Remarkably, a right localization was observed most often in sporadic PHEO, whereas in patients with hereditary PHEO, these were mostly located in the left adrenal. No differences in pathways to discovery, associated symptoms, or functionality were found between sporadic and hereditary PHEO. The median duration of follow-up was shorter in sporadic vs hereditary disease (3 (0.6–17) vs 8.5 (2–26) years; *P* = 0.03). At follow-up, most of the patients were disease-free (23 (85%) and 7 (87%) in sporadic and hereditary PHEO, respectively). Recurrence occurred in one (3.7%) patient with sporadic disease 13 years after the initial diagnosis, while three (37.5%) patients with hereditary PHEO presented with a new disease (new localization of the tumours) after a median of 7 (4-12) years; two (6.25%) patients with sporadic PHEO are still having active disease (one with metastatic disease). Unfortunately, two patients with sporadic disease are now deceased, without having information about the cause of death (Table 2).

**Table 2:** Patient and tumour characteristics in sporadic vs hereditary PHEO. Data are presented as mean ± s.d., number (percentages), or median (absolute range).

*n* = 35	Sporadic (*n* = 27)	Hereditary (*n* = 8)	*P* value
Age (years)	55 ± 16.9	44.8 ± 17.8	0.152
Gender	17 F, 10 M	3 F, 5 M	0.246
BMI (kg/m^2^)	26.5 ± 4.8	24.6 ± 3.9	0.368
Localization			
Right	16 (59.3%)	1 (12.5%)	0.001
	Left	11 (39.8%)	4 (50%)	0.002
	Bilateral	0	3 (37.5%)	0.033
Functionality^a^	26/26 (100%)	7/7 (100%)	1
Multiple tumours at diagnosis	0	3 (37.5%)	0.033
Metastatic disease	3 (8.8%)	1 (12.5%)	0.910
Pathway to discovery			
Symptomatic	15 (55.6%)	4 (50%)	1
Incidentally/genetic screening	12 (44.4%)	4 (50%)	1
Symptoms			
Classic triad	13/24 (54.1%)	2/5 (40%)	0.997
Non-specific	6 (22.2%)	2 (25%)	1
Tumour location-related	1 (3.7%)	0	0.875
CV events	10 (29%)	4 (50%)	0.398
MN levels (*n* < 350 µg/24 h)	2584 (range 63–32525)	541 (range 230–4518)	0.596
Number of patients with MN levels above ULN	15/24 (62.5%)	2/4 (50%)	1
xULN^b^	12.9	4.1	0.400
NMN levels (*n* < 650 µg/24 h)	2684 (range 326−18813)	3195 (range 926–9841)	1
Number of patients with NMN levels above ULN	19/24 (79.2%)	2/4 (50%)	0.253
xULN^b^	6.8	6.5	0.947
D levels (*n* < 500 µg/24 h)	258 (range 62–1549)	594 (range 270–6263)	0.269
Number of patients with D levels above ULN	1/18 (2.1%)	1/4 (25%)	0.021
xULN^b^	0.7	3.8	0.027
Tumour diameter (mm)	53 (range 17–200)	52 (range 20–75)	0.714
SDHB staining (− negative; + positive; +/− weak)	8 −	1 −	
	5 +	4 +	
	2 +/−	0 +/−	
	12 Not known	3 Not known	

^a^Data only available for subset of patients; ^b^Calculated only for those with functional tumours.

D, dopamine; MN, metanephrine; NMN, normetanephrine; NA, not applicable; xULN, times upper limit of normal.

### Sporadic vs hereditary PGL

Patients with hereditary PGL had a younger age at diagnosis than those with sporadic disease (*P* = 0.001). More sporadic PGL were located right-sided compared to hereditary PGL, although this difference was not significant (*P* = 0.071). There were no important differences in pathway to discovery, symptoms, metanephrine levels, or tumour diameter between sporadic and hereditary PGL. The median duration of follow-up was equal between the two groups (4 (1–12) vs 4 (1–34) years). At follow-up, most of the patients were disease-free (6 (67%) and 9 (56%) in sporadic and hereditary PGL, respectively). One patient with sporadic PGL had a recurrence after 10 years, while in patients with hereditary PGL, we observed one patient with new disease, three with new disease plus recurrence, and one with recurrence after a median time of 10 (2–18) years from the initial diagnosis. Further, active disease is documented in one patient with sporadic and seven with hereditary PGL (one patient with metastatic disease). Two patients with sporadic disease are now deceased, without having information about the cause of death (Table 3).

**Table 3:** Patient and tumour characteristics in sporadic vs hereditary PGL. Data are presented as mean ± s.d., number (percentages), or median (absolute range).

*n* = 25	Sporadic (*n* = 9)	Hereditary (*n* = 16)	*P* value
Age (years)	55 ± 16.5	32 ± 13	0.001
Gender	4 F, 5 M	9 F, 5 M	0.688
BMI (kg/m^2^)	28 ± 4.5	22 ± 4	0.010
Localization			NA
	5 (55%) HNPGL	7 (43%) HNPGL	
	1 (11%) Thoracic	2 (12.5%) Abdominal	
	2 (22%) Abdominal	4 (25%) Pelvic	
	1 (11%) Abdominal + Pelvic	2 (2.5%) HNPGL + Thoracic	
		1 (6.25%) HNPGL + Abdominal	
Lateralization			
Right	7 (77.7%)	5 (31.2%)	0.071
	Left	2 (22.2%)	8 (50%)	0.613
	Bilateral/central	-	3 (18.7%)	NA
Functionality^a^	3/5 (60%)	7/12 (58%)	0.605
Multiple tumours at diagnosis	1 (11%)	5 (31.2%)	0.364
Metastatic disease	1 (Synchronous)	1 (Metachronous)	0.500
Pathway to diagnosis			
Symptomatic	5 (55.5%)	8 (50%)	0.881
Incidentally/genetic screening	4 (44.4%)	8 (50%)	0.057
Symptoms^a^			
Classic triad	1/7 (14.3%)	4/12 (33.3%)	0.631
Non-specific	0	7/15 (46.6%)	0.067
Tumour location-related	3 (33.3%)	4 (25%)	0.142
CV events	3 (33.3%)	0	0.037
MN levels(*n* < 350 g/24 h µg/24 h)	223 (range 211–400)	145 (range 41–271)	0.126
Number of patients with MN levels above ULN	1/4	0/11	0.500
xULN^b^	1.1	-	NA
NMN levels (*n* < 650 µg/24 h)	719 (range 406–1400)	648 (range 126–8694)	0.659
Number of patients with NMN levels above ULN	2/4 (50%)	5/12 (41,7%)	0.997
xULN^b^	2.15	5.82	0.651
D levels (*n* < 500 µg/24 h)	233 (range NA)	314 (range 203–741)	0.600
Number of patients with D levels above ULN	0/1	2/9	0.054
xULN^b^	-	1.34	NA
Tumour diameter (mm)	28 (range 15–40)	42 (range 11–110)	0.164
SDHB staining (–negative;+positive; +/– weak)	3 +	2 +/–	
	0 –	8 –	
	6 Not known	6 Not known	−

^a^Data only available for subset of patients; ^b^Calculated only for those with functional tumours.

D, dopamine; MN, metanephrine; NMN, normetanephrine; NA, not applicable; NED, no evidence of disease, xULN, times upper limit of normal.

### Tumour characteristics in hereditary and apparently hereditary PPGL

For documented hereditary PPGL, disease characteristics per affected gene are given in Table 4. In the 10 patients with *SDHD*-related disease, most (10/13) tumours were HNPGL with only 2 thoracic and 1 abdominal PGL, and 1 PHEO. Half of patients with *SDHD*-related disease presented with multiple tumours at diagnosis. For *SDHB*, tumours were mostly (6/8) pelvic or abdominal PGL, with only one PHEO and one HNPGL. The algorithm for genetic testing (Lenders *et al.* 2014) did not fit in 3 out of the 24 patients with hereditary disease as we observed one case of metastatic disease (arteria renalis invasion) in a patient with a *VHL* mutation and positive *SDHB* staining in 2 *SDHD*-related PPGL. Also, three patients presented with disease characteristics suggestive for hereditary PPGL (two aged < 45 years and one with two synchronous PGL at diagnosis) but with negative genetic testing.

**Table 4:** Tumour characteristics per affected gene in hereditary PPGL.

Gene	Localization	Metastatic	Secretion pattern	SDHB staining^a^
*SDHD* (10)	1 PHEO, 6 HNPGL	1	Noradrenergic	4 −
	2 HNPGL + Thoracic			2 +/−
				4 No info	1 HNPGL + Abdominal
	*SDHB* (8)				1 PHEO	0	Noradrenergic	5 −
1 HNPGL				3 No data
2 Abdominal				
4 Pelvic				
*VHL* (2)	2 PHEO	1	No data	No data
*RET* (2)	2 PHEO	0	Adrenergic	+
*MAX* (1)	PHEO	0	Noradrenergic	+
*NF1* (1)	PHEO	0	Adrenergic	+

^a^SDHB staining (− negative; + positive; +/− weak).

## Discussion

This study shows that there were no important differences in pathways to diagnosis, clinical, or biochemical phenotype of sporadic vs hereditary PPGL in a cohort of Belgian patients. Genotype–phenotype correlations identified in our cohort are largely similar with those from the literature, although in one patient with multiple PGL locations, no pathogenic germline mutation could be identified. Also, the algorithm for genetic testing (Lenders *et al.* 2014) did not fit in 3 out of the 24 patients with a documented hereditary disease which confirms that a multiple gene-panel to screen these patients should be considered clinical state-of-the-art.

In this retrospective cohort, we observed a predominance of PHEO cases but more than one-third of our patients presented with a PGL, which is within the range reported in other series (Amar *et al.* 2005, Leung *et al.* 2021). Further, 23% of patients with PHEO showed a hereditary predisposition, whereas for PGL, this was 64%. Indeed, it is known that PHEO are more frequently sporadic than PGL (Karasek *et al.* 2010, Hensen *et al.* 2011). Among all genetically tested PPGL patients, 40% of patients carried a genetic predisposition which corroborates literature findings in which up to 40% of the PPGL are reportedly caused by a germline mutation, with mutations in *SDHD*/*SDHB*/*VHL* genes being most common (Boedeker *et al.* 2007, Karasek *et al.* 2010, Eisenhofer *et al.* 2011, Lenders *et al.* 2014, Muth *et al.* 2019). In our cohort, *SDHD* mutations were most frequent, followed by *SDHB*. It should be noted, however, that 7 out of our 67 patients were not genetically tested and that the gene panel used at the time of screening had a variable composition.

Remarkably, we observed a predominant right/left localization in sporadic/hereditary PHEO (and similar but non-significant trends in PGL), which is an aspect that was not described earlier. We did not find any significant correlation between tumour localization (right/left) and tumour diameter, metanephrine levels, occurrence of CV events, or pathway discovery (data not shown) and suppose that these differences in lateralization are coincidental rather than of clinicopathological importance.

The distribution of incidentally/genetically screened vs symptomatically discovered PPGL was equal among both sporadic and hereditary PPGL. These findings emphasize the non-specific character of the symptoms in these diseases, along with an increasing rate of imaging-based diagnosis. In other studies, the incidental or screening-based finding of the tumour was the first mode of diagnosis (Falhammar *et al.* 2018, Gruber *et al.* 2019, Cvasciuc *et al.* 2020). We found that the classic triad was most frequently reported in patients with sporadic PHEO, while in hereditary PHEO, symptoms were more often non-specific but without statistically significant differences. The frequency of classic triad was higher in our cohort than in other studies, where it was reported in 10–28% of cases (Kopetschke *et al.* 2009, Falhammar *et al.* 2018, Geroula *et al.* 2019, Cvasciuc *et al.* 2020)*.* We also observed more prevalent CV events in patients with PHEO as compared to PGL. Although this might result from diagnostic bias and the higher number of functionally active tumours in the former, future research should address if patients with PHEO really are at increased risk of CV events. In our cohort, only in one patient with Takotsubo cardiomyopathy occurring shortly after abdominal manipulation, a direct link with the PHEO could be presumed.

As expected, patients with hereditary PPGL presented more often with new or recurrent disease (8/24) than those with sporadic PPGL (2/36). This justifies a life-long follow-up for patients with hereditary PPGL. However, one patient with a sporadic PHEO presented with a recurrence after 13 years, supporting the European Society of Endocrinology guideline proposing lifelong follow-up for all high-risk patients (i.e. young patients, or with large tumours, or with PGL) (Plouin *et al.* 2016).

Regarding genotype–phenotype correlations in those patients with hereditary PPGL, we found that anatomical distribution of the tumours and secretion pattern according to the mutated gene largely correspond to the literature. Specifically, patients with *SDHD* mutations presented mostly with HNPGL (9/13), a presentation also described in a Dutch cohort (Hensen *et al.* 2011). Most *SDHD*-related PPGL are benign, which is confirmed by our findings*.* One out of 10 patients with *SDHD* mutation had metastatic disease after a median follow-up duration of 12.1 years. In patients with pathogenic *SDHB* mutations, PHEO occurrence and abdominal/pelvic PGL (7/8) were more frequently observed, also as previously reported (Muth *et al.* 2019). In contrast to other reports where the malignancy rate was relatively high (van Hulsteijn *et al.* 2012, Tufton *et al.* 2017, Main *et al.* 2020), none of these eight patients developed malignant disease, although the median follow-up was only 7 years.

Tumour *SDHB* expression as determined by immunohistochemistry largely corresponded with the literature on patients with hereditary disease (van Nederveen *et al.* 2009)*.* However, in eight PHEO patients with negative SDHB immune-staining, no pathogenic mutation in SDHx-genes could be demonstrated. In one of these patients, a somatic SDHB mutation was found, three patients had metastatic disease, and four did not have a hereditary phenotype based on clinical and anamnestic observations. Although reported sensitivity and specificity of SDHB immunohistochemistry for detecting SDHx germline mutations range between 90% and 100% and 60% and 97%, respectively (van Nederveen *et al.* 2009, Evenepoel *et al.* 2015), our findings suggest a more variable degree of reliability. This variability could be related to somatic SDHx mutations (not routinely tested at our centre), unknown germline pathogenic variants, or to non-sensitive or non-specific immunohistochemical staining.

Apparently hereditary PPGL with negative genetic screening represent a challenge for the clinician. In our cohort, two out of three PHEO with metastatic disease had negative *SDHB* immunostaining and noradrenergic phenotype, but we could not identify a pathogenic *SDHx* gene mutation. VHL was not tested due to the lack of availability of this gene sequencing at the time of diagnosis; however, generally *VHL* mutations are not associated with negative *SDHB* staining (van Nederveen *et al.* 2009, Evenepoel *et al.* 2015). These two patients had no family history of PPGL but were under 45 years old. In these apparently hereditary cases, there are some possibilities. First, the disease is related to pathogenic *SDHx* mutations escaping detection by DNA sequencing (deleterious mutations in untranslated, intronic or promoter regions, low-level mosaicism in lymphocytes) or by epigenetic silencing of *SDHx* genes. Second, the spectrum of genetic mutations involved in PPGL development is incompletely known and continuously evolving so we cannot exclude a germline mutation which was not tested or discovered yet. For instance, the FH gene in which pathogenic mutations can also lead to the development of PPGL is not yet tested in all centres or patients. Third, it could be that these apparently hereditary PPGL are caused by a somatic mutation with an aggressive potential (e.g. *ATRX, TERT* mutations).

In conclusion, we found that patients with PGL more frequently have a hereditary predisposition than those with PHEO, contributing to the fact that diagnosis is generally made earlier in PGL. Although diagnosis in both PHEO and PGL was mostly due to related symptoms, patients with PHEO more often presented with CV comorbidities than those with PGL. The only minor differences between sporadic and hereditary PPGL and sometimes discordant genotype–phenotype correlations challenge the management of these diseases. Knowledge of clinical behaviour and evolution in tumours with somatic mutations is still in progress and more detailed tumour genotype–-phenotype research in sporadic PPGL should be done. Patients with apparently sporadic PPGL but with aggressive behaviour should be tested for somatic mutations. In addition, genetic diagnostic laboratories should extend the gene panel for PPGL with other newly discovered genes such as *FH, EPAS, MDH2, SLC25A11,* and others, at least until whole exome/genome sequencing is routinely available. Also, the inclusion of these genes should be accompanied by the development of clinical guidelines for follow-up and management of these patients in whom germline variants will be identified.

## Declaration of interest

All authors have nothing to disclose.

## Funding

This study did not receive any specific grant from any funding agency in the public, commercial or not-for-profit sector.
